# Case of Malignant Tumor of Eight or Ten Years Standing, Cured after Two Years, by a Strict Diet of Bread and Mills, with Remarks

**Published:** 1849-12

**Authors:** 


*Case of Malignant Tumor of eight or ten years standing, cured after two years, by a strict Diet of Bread and Milk, with remarks.* By H. J. Bowditch, M. D., of Boston, Mass.

In accordance with your request, I copy for your Journal my notes of the very interesting case of Dr. Twitchell. I obtained them from him during my late visit to the Granite State, and he kindly allows me to publish them. Every medical man, I presume, is somewhat acquainted with Dr. T. He is one of the most noted of our New England surgeons. His circuit has a diameter of fifty miles—and he has always, even while suffering from the local disease I shall endeavor to detail, been able to drive his hundred miles, if necessary, in the twenty-four hours, and in his own carriage, over the hills of his native state. The medical history of his *life* is extremely interesting. I shall therefore give that, very briefly, before entering upon the consideration of his local disease.

1st. Carcinoma has appeared in his family. His grandmother died of cancer of the mamma; his sister of a scirrhous pylorus. These are all the data of his hereditary tendencies that bear upon our main topic.

2d. In very early life, Dr. T. was in delicate health. As a youth, he was stronger, and was among the foremost in all athletic sports. While at college, he became dyspeptic; had icterus, with enlarged liver, &c.; subsequently, he passed gall-stones. Whilst pursuing the studies of his profession, he began to suffer from asthma, and for about 20 years, was very much subject to violent attacks of it, causing orthopnœa, &c. During all this period, he ate animal food very freely, three times daily, and digested it easily, whereas vegetable food caused dyspeptic difficulties. Being induced, owing to a severe acne of the face, to abandon this course, he gave up, for nine years, the use of meat. From the period at which he first abandoned meat, he has never had an attack of asthma, and Dr. T. considers these two facts related to each other, as cause and effect. Moreover, vegetable food was soon easily borne. After the nine years of vegetable regimen, he began gradually to resume the use of the milder kinds of animal food, such as poultry, and somewhat of the more solid meats, until two years since, when he commenced the very rigid diet, which will be described when treating of his local disease, which is the more immediate object of this paper. Finally, I will state, as indicative, perhaps, of the tendencies of the cutaneous system to morbid action, that about four years ago, he had a papular eruption lasting six weeks, and, likewise, that very many years ago, he had a wart-like tumor on the scalp, which disappeared under the use of creosote, externally applied.

3. The local disease, the course and result of which, I present as the chief object of interest, commenced eight or ten years since, as a small but hard tumor at the internal angle of the right eye. When first noticed, it was about as large as a mustard seed, and not painful. He occasionally touched it, and had some suspicion that it might eventually prove to be of a malignant character. It was imbedded in the substance of the cutis, and from the first, seemed very slowly to augment in size. At times he *thought* he felt some lancinating pains in it, which radiated to the brow. It however did not interfere with the functions of the lachrymal ducts, &c. About 1843 the tumor had become nearly as large as a pea, and a tendency to the formation of a scab was observed. He then was induced to try some local applications, and frequently, until 1845, used "Jenning's

Ointment."* This would remove the scab, and displayed three small lobes from which exuded a little purulent fluid. At first the morbid growth seemed lessened by this and other milder applications, but no permanent effect was produced. At times the discharge ceased, but only to return again, and the tumor gradually lost its trilobed aspect. It was at this period quite conspicuous to every bystander.

August, 1845, Dr. Geo. Hayward, of this city, removed the major part of it with the scalpel. For a short time, the wound seemed doing well; but finally, it did not heal, and two months afterwards it was operated on again, and nitrate of silver was applied. Meanwhile, however, there had been experienced much local pain. It was deeper seated, less transitory, and radiated towards the brow and cheek. Sometimes it was severe enough to awaken him at night, and was worse usually after long rides.

The applications during 1846-7 were chiefly of a very simple character; cold cream, preparations of zinc, &c., and once the iodide of lead. All active application caused inflammation of the conjunctiva. The tumor continued to augment slightly, and in the spring of 1847, it presented to my eye a decidedly malignant appearance. It was an ulcer about the size of the top of the finger, with ragged, hard, elevated edges, and the irritation from the discharge caused the patient frequently to apply his handkerchief to the part. At night it caused a glueing of the lids, and a discharge on the side of the nose. I certainly believed, and Dr. T. tells me that he thought, at that time, that the disease would gradually augment and involve the eye—and he had determined, if necessary, to have this organ extirpated. His general health, as it has been already stated, continued good; but, when not actively employed, the mind was somewhat depressed at the prospect before him. At the meeting of the American Medical Association in Philadelphia, May, '47, he consulted several of the eminent men whom he met. I believe I may say, that all regarded it as a disease of a most serious nature, although some thought it might be cured by local applications, and others advised a further operation.

Dr. T. returned home discouraged, and he decided to give up all use of medicines internally, or of external applications, but to try a course of the most rigid diet. Starting from a theory that malignant diseases arise from the fact that we take too much carbon into our system, he determined to live, from that time, upon a bread and milk diet, and if at the end of some months he did not find any diminution in the disease, he still determined to use nothing but bread and water. Since his return from Philadelphia, he has strictly adhered to the bread and milk. He has used three times daily, from ℥iv. to ℥vi. of cream or the richest milk, and same quantity of either white or brown bread. He continues that diet still.

The results upon the *local disease* have been as follows:—The pains in the part were lessened almost immediately. The purulent discharge very soon began to lessen, and in two or three months, it was evident that the disease was not augmenting. During the following winter the improvement was more decided. In the spring of 1848, being obliged to ride over dusty roads, to great distances, the eye was more irritated. Nevertheless, he felt, and his friends assured him, that the diseased part was

* Mackenzie on the Eye.

really lessening and tending towards a cure. Since that period, a steady improvement has taken place. The ulcerated mass which was so perceptible to me, two years since, has wholly gone, and now, (August, 1849) I can discover no difference between the angles of the two eyes, save that in the right one there is a minute white spot, about a line in diameter, looking like a cicatrix. It is not harder than the adjacent parts, and had I not known of the existence of previous disease, I should not have noticed even this. There is no discharge, no pains, and a perfect cure seems to have been accomplished, of a disease that had been existing for about ten years, in a patient aged 68 years.

The effects of this rigid diet upon the constitution, as a whole, are interesting.

In his mental state, Dr. T. thinks he has been much less irritable than when he was *omnivorous*.

He had, at one time, an attack of vertigo, (to which, however, he has been always liable), and, finding that he was *growing corpulent* under the diet, he, for a time, took less of it.

He has always been as strong as when indulging in a more generous diet.

He has been able to breathe better, having had less tendency to dyspnœa.

His digestion has been good, but with a slight tendency to costiveness.

His organs of circulation have been unaffected.

Renal excretion, for years, a little disturbed, as is not unfrequently the case in persons of his age.

Finally, Dr. T. presents to my mind the picture of a hale, robust man, in perfect health, so far as one can perceive, and but slightly touched by the influence of his many years of honorable and successful labor.

*Reflections upon Dr. T.'s Case.*—The most important topic involved in the foregoing record, is the restoration to health from what seemed to be malignant disease, and that this result followed the strict diet of bread and milk for two years.

*Second.* The cessation of asthmatic difficulties, after they had troubled the patient for twenty years, and that this cure likewise followed the change of diet—from an almost strictly animal diet to one quite the reverse, viz., strictly vegetable.

*Third.* Some readers may ask if these two cures are not merely examples of the "*post-hoc;*" and they may deny that there is any complete evidence of the "propter hoc." I consent to the doubt, for it has entered my own mind. Nevertheless, if they are mere coincidences, they are pregnant with important suggestions. I confess that, in my own practice, I have never met with any cases so significant of the power that diet, simply and heroically used, has to *re-organize* a man.

*Fourth.* Dr. T.'s case becomes interesting as an evidence of the power of a man to subject his body to strict rule. In this epicurean age, it is quite refreshing to find one who "eats to live, and does not live to eat." A worthy professional brother of this city, said, when the case was related to him, "It might certainly be a question whether life were desirable under such a regimen!" I honor a hero wherever I find him, and the heroism of Dr. T., in undertaking and pursuing this course so long, merely in consequence of a theory, excites in me the greatest delight. In this sceptical, unbelieving era, I like to see any one having *faith*. Whether the theory was correct or not, it matters little—the fixed will of its follower arouses my enthusiasm; and this brings me to another topic of interest.

*Fifth.* The theory which governed Dr. T.—was it correct? I confess that I am unable to solve the question; I merely suggest it. Some, whom I consider as our ablest animal chemists, think that it was by the process of starvation, as described by Liebig,* that the cure was wrought. It seems to me that this cannot be the true explanation—for Dr. T. has always been stout, and it will be remembered that at one time he actually gained flesh under the diet.—*Charleston Med. Jour. and Review.*

* Animal Chemistry, Cambridge ed., p. 25. 1842.

